# Principles of agonist recognition in Cys-loop receptors

**DOI:** 10.3389/fphys.2014.00160

**Published:** 2014-04-24

**Authors:** Timothy Lynagh, Stephan A. Pless

**Affiliations:** Department of Drug Design and Pharmacology, Center for Biopharmaceuticals, University of CopenhagenCopenhagen, Denmark

**Keywords:** ion channels, Cys-loop receptors, nicotinic acetylcholine receptors, GABA-A receptors, glycine receptors, serotonin receptors, ligand recognition, GluCl

## Abstract

Cys-loop receptors are ligand-gated ion channels that are activated by a structurally diverse array of neurotransmitters, including acetylcholine, serotonin, glycine, and GABA. After the term “chemoreceptor” emerged over 100 years ago, there was some wait until affinity labeling, molecular cloning, functional studies, and X-ray crystallography experiments identified the extracellular interface of adjacent subunits as the principal site of agonist binding. The question of how subtle differences at and around agonist-binding sites of different Cys-loop receptors can accommodate transmitters as chemically diverse as glycine and serotonin has been subject to intense research over the last three decades. This review outlines the functional diversity and current structural understanding of agonist-binding sites, including those of invertebrate Cys-loop receptors. Together, this provides a framework to understand the atomic determinants involved in how these valuable therapeutic targets recognize and bind their ligands.

## Diversity, physiological importance, and fundamental architecture

Numerous physiological processes rely on the rapid conversion of extracellular chemical signals into electrical signals at the cell membrane. This is predominantly mediated by ligand-gated ion channels (LGICs), membrane-embedded ion channels that are allosterically activated upon binding of an agonist, usually a neurotransmitter. A large family of LGICs is that of the pentameric Cys-loop receptors, sometimes referred to as pentameric ligand-gated ion channels, as not all members of this protein family contain the eponymous Cys-loop. These receptors are broadly divided into excitatory and inhibitory receptors, based on the permeability of the integral ion channel to cations or anions, respectively. As regards the human nervous system, passage of sodium and calcium through excitatory nicotinic acetylcholine, and serotonin type 3 receptors (nAChRs and 5-HT_3_Rs) depolarizes the membrane, whereas chloride permeability through inhibitory GABA type A and glycine receptors (GABA_A_Rs and GlyRs) generally serves to hyperpolarize the membrane potential and thereby decrease cellular excitability. Activation of nAChRs by acetylcholine mediates vital neuromuscular and autonomic signals (Langley, 1901; Bennett, 2000) and the importance of nAChRs in the brain is highlighted by the well-documented effects of nicotine on cognition (Levin, 2002). 5-HT_3_Rs mediate several effects of serotonin on maturation of glutamatergic and GABAergic networks (Engel et al., 2013) and are targets for widely used anti-emetic drugs (Lummis, 2012). Humans also express transcripts of a unique Cys-loop receptor isoform that, when expressed recombinantly, forms zinc-activated cation channels, although little is known about its function (Davies et al., 2003). Regarding the inhibitory receptors, some overlap occurs in the expression patterns of GABA_A_Rs and GlyRs. GABA_A_Rs are the primary mediator of inhibitory signals in the brain (Sigel and Steinmann, 2012), and pharmacological enhancement of GABA_A_Rs by benzodiazepines and anesthetics underlies widely used anxiolytic therapies (Korpi and Sinkkonen, 2006) and general anesthesia (Zeller et al., 2008), respectively. Inhibitory GlyR function, on the other hand, appears to dominate in the spinal cord and brain stem, regulating different motor and sensory functions, including pain, and GlyRs are also involved in processing auditory and visual signals (Lynch, 2009).

Each of the above receptors is further divided into subtyes, composed of varying combinations of different subunit isoforms. In humans, there are five known GlyR isoforms (α1 through α4 and β; Harvey et al., 2000) and five 5-HT_3_R isoforms (named A to E; Lummis, 2012). By contrast, nAChR and GABA_A_R isoforms show a far greater degree of diversity: 17 different isoforms are known for nAChRs [nine α (termed α1, α2, α3, and so on), four β, one γ, one δ, and one ε isoforms] and 19 different isoforms for GABA_A_Rs (six α, three β, three γ, one each of δ, ε, π, θ, and three ρ isoforms; Collingridge et al., 2009). Although some subtypes are homomeric pentamers, the majority of native Cys-loop receptors are heteromers. Together with the significant isoform diversity, this results in a large number of possible permutations (although the most prominent stoichiometry in the brain is 2xα1, 2xβ2, and 1xγ2; Olsen and Sieghart, 2008).

However, the true diversity of the Cys-loop receptor family is only realized when invertebrate and bacterial members are considered. These include, in addition to nAChR-like and GABA_A_R-like receptors, cation channels gated by betaine (Peden et al., 2013), GABA (Ranganathan et al., 2000) primary amines (Zimmermann and Dutzler, 2011), and pH (Bocquet et al., 2007); anion channels gated by glutamate (Cully et al., 1994), histamine (Zheng et al., 2002), dopamine, serotonin, tyromine (Ringstad et al., 2009), and pH (Schnizler et al., 2005); and acetylcholine-binding proteins (AChBPs) that resemble the extracellular half of nAChRs and serve to buffer excessive transmitter at the molluscan synapse (Smit et al., 2001). Incidentally, it is lower organisms that have contributed the Cys-loop receptors most amenable to structural methods, and the resolution with which we now view receptor structure is based on X-ray crystallographic structures of two bacterial cation channels referred to as ELIC and GLIC (gated by primary amines and protons, respectively; Hilf and Dutzler, 2008, 2009; Bocquet et al., 2009; Zimmermann and Dutzler, 2011; Spurny et al., 2012), the α glutamate-gated chloride channel from *Caenorhabditis elegans* (Hibbs and Gouaux, 2011; α GluCl, or GLC-1; Beech et al., 2010) and AChBPs from *Lymnaea stagnalis* (Brejc et al., 2001) and *Aplysia californica* (Hansen et al., 2005). These have superceded electron micrographic data on nAChRs from the ray *Torpedo marmorata* (e.g., Unwin, 2005) that consolidated early notions of receptor structure.

**Figure 1:**
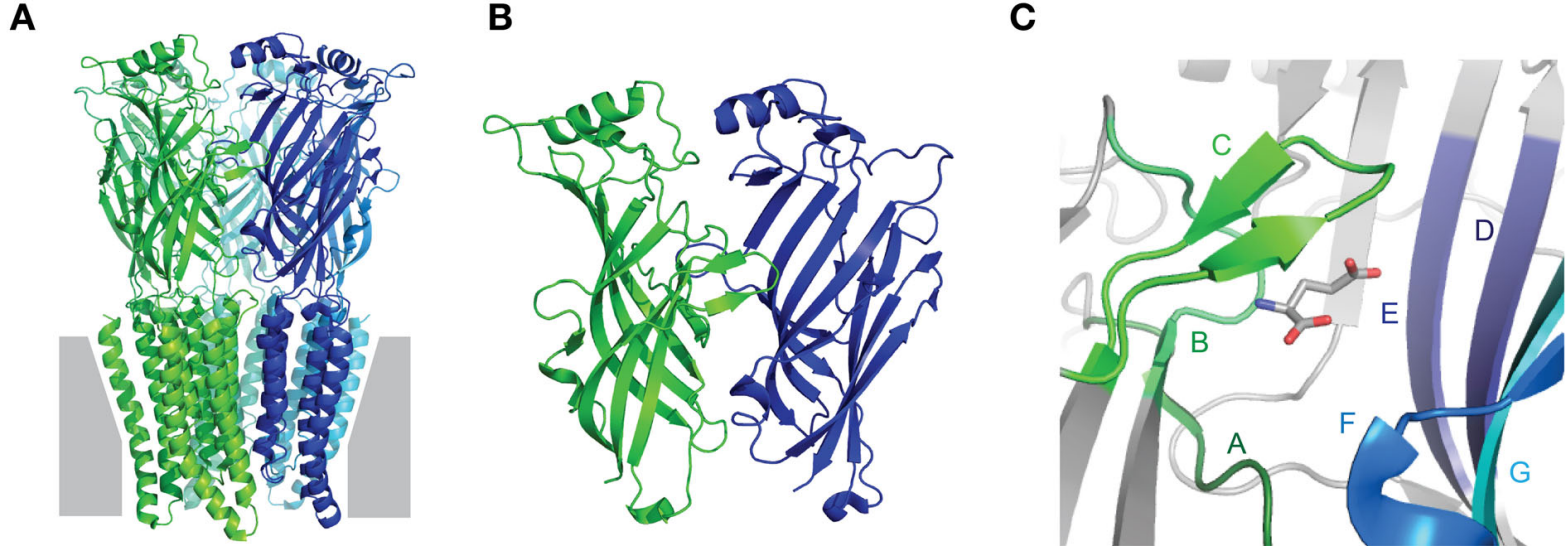
**Fundamental Cys-loop receptor architecture. (A)** Pentameric Cys-loop receptor, viewed from within the membrane (gray) plane. Each of the five subunits is indicated by a different color and contains an extracellular N-terminal extracellular domain (ECD) consisting of two β-sheets and a membrane-spanning domain comprised of four α-helices. **(B)** Magnified view of the interface of adjacent ECDs: one subunit in green, one in blue. The outer β-sheet of the green and the inner β-sheet of the blue form the principal and complementary faces, respectively, of the agonist-binding site. **(C)** Magnified view of the agonist-binding site, showing agonist-binding loops A–C of the principal face and D–G of the complementary face. All images are based on the glutamate-bound *Caenorhabditis elegans* α GluCl crystal structure, Protein DataBase reference 3RIF (Hibbs and Gouaux, 2011).

Collectively, the crystal structures confirm early biochemical studies, in that Cys-loop receptors are pentamers in which each subunit contains an extracellular domain (ECD), consisting of 10 consecutive strands arranged in two β-sheet cores, followed by four membrane-spanning helices (M1–M4), ending in a small extracellular C-terminal tail (Figure 1A). The agonist-binding site is situated at the interface of adjacent ECDs (Figure 1B). The principal face of the agonist-binding site comprises three loops (“A–C”) from the outer β-sheet of one subunit, and the complementary face comprises three β-strands and one loop (“Loops D–G”) from the inner β-sheet of the adjacent subunit (Figure 1C). The five subunits are arranged in five-fold symmetry, such that a central ion channel is formed by the apposition of all M2 helices. Opening of the ion channel occurs as a result of agonist-induced conformational changes in the ECD, which in turn trigger conformational changes within the ion channel, in an allosteric process often termed ligand-gating or agonist-induced activation (Twyman and Macdonald, 1991; Miller and Smart, 2010). It is important to note that, depending on which isoforms are present, not all subunits contribute equally to agonist binding or subsequent channel gating, so the number of binding sites can vary among different receptors. Some nAChRs have been reported to open in response to a single bound agonist (Andersen et al., 2013) or even in the absence of any ligands (Jackson, 1986; Purohit and Auerbach, 2009). However, most Cys-loop receptors are thought to require binding of 2–3 agonist molecules for most efficient activation (Sine et al., 1990; Beato et al., 2002; Rayes et al., 2009; Harpsoe et al., 2011).

**Figure 2:**
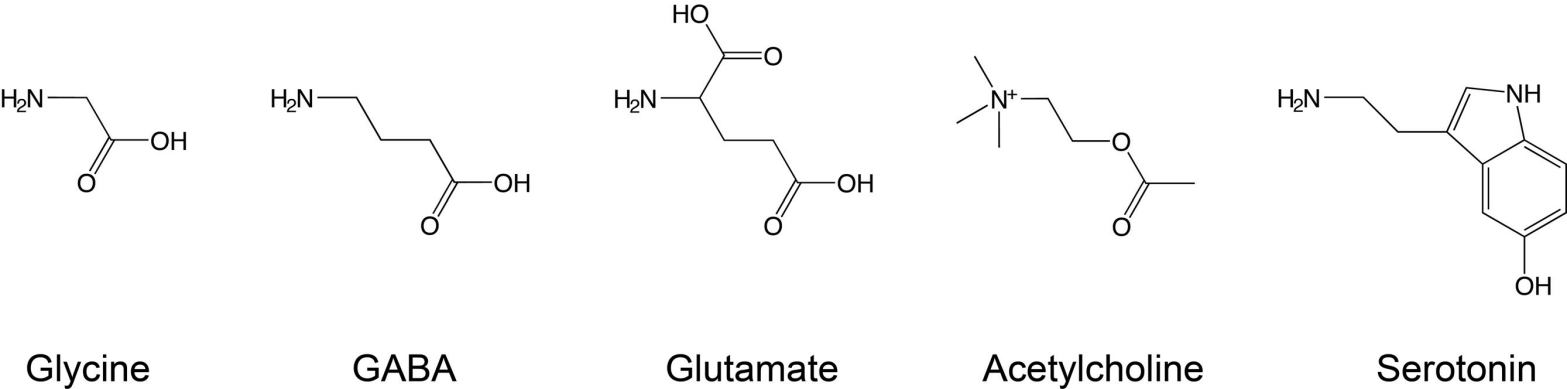
**Conventional Cys-loop receptor agonists.** Glycine, GABA, and glutamate are neurotransmitters that bind to inhibitory receptors, while serotonin and acetylcholine bind to excitatory receptors. Note the different electrical state of these agonists under physiological conditions: glycine and GABA are zwitterions, while acetylcholine and serotonin carry a single positive charge and glutamate a net negative charge.

Ligands that induce channel opening are termed agonists, although some agonists induce channel opening with poor efficiency and are therefore termed partial agonists. The five classical neurotransmitter agonists considered in this review are shown in Figure 2, illustrating their vaguely linear structure, with polar N- or O-containing termini. Other ligands, termed competitive antagonists, bind in the agonist-binding site and can elicit conformational changes but prevent binding of agonists and thus channel activation. As this review focuses on the primary determinants of ligand-receptor interactions at the agonist-binding site, we will continuously refer to “recognition” of “agonists” for consistency.

## Tracing the recognition of diverse agonists to subtle molecular differences

**Figure 3:**
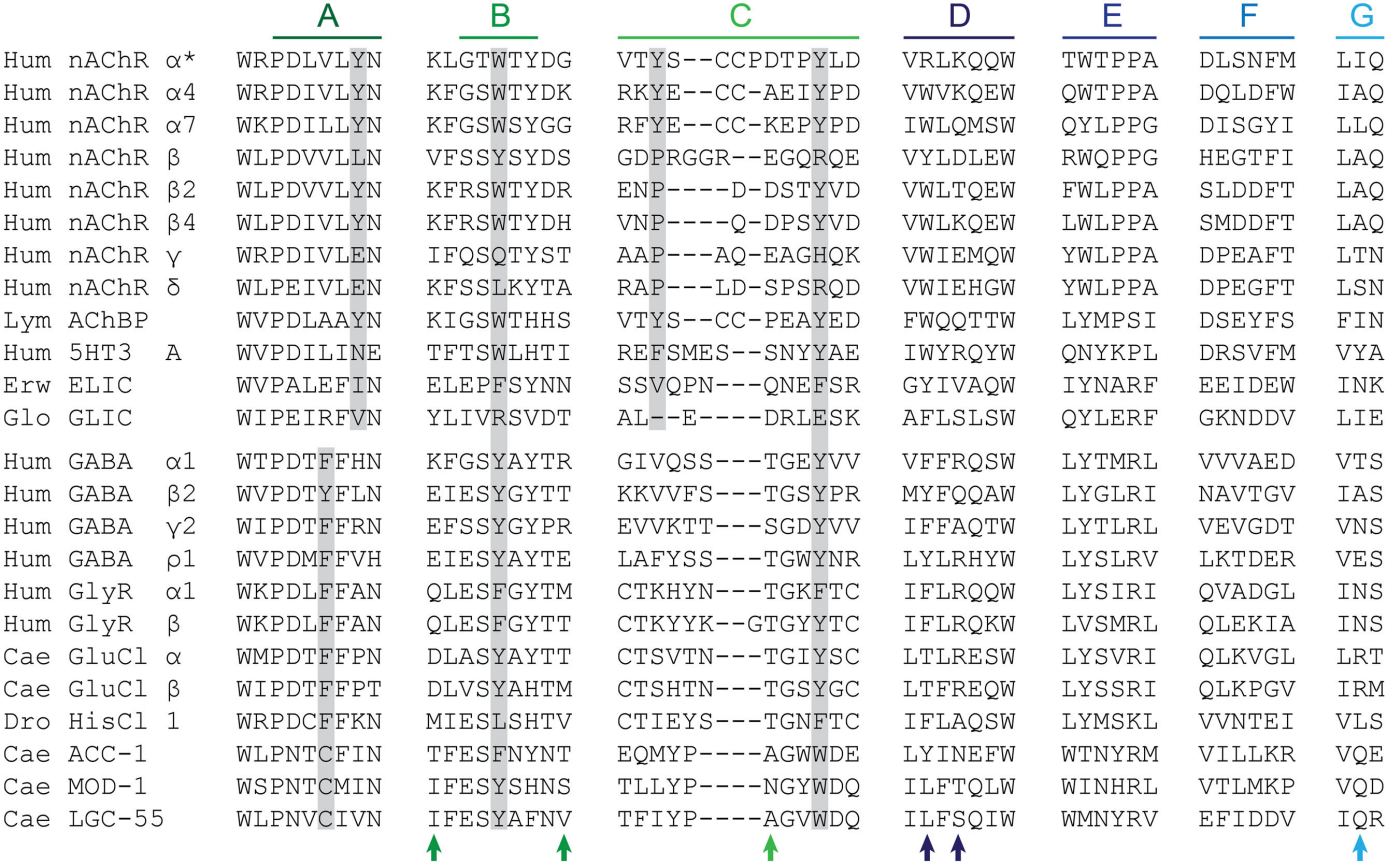
**Amino acid sequence alignment.** Only side chains in agonist-binding loops are shown, plus small segments abutting Loop A and Loop B. *Hum*, human; *Lym*, *Lymnaea stagnalis*; *Erw*, *Erwinia chrysanthemi*; Glo, *Gloeobacter violaceus*; *Cae*, *Caenorhabditis elegans*; *Dro*, *Drosophila melanogaster*. ^*^Refers to nAChR isoform 1, which is up-regulated in muscle but less abundant in humans than isoform 2, which contains a 25 amino acid insert in Loop D (Beeson et al., 1990; Talib et al., 1993). While ELIC gates in response to primary amines, GLIC is a proton-gated cation channel. HisCl 1 (Zheng et al., 2002), ACC-1 (Putrenko et al., 2005), MOD-1 (Ranganathan et al., 2000), and LGC-55 (Ringstad et al., 2009) are histamine, acetylcholine, serotonin, and tyromine-gated chloride channels, respectively. Gray boxes indicate conserved aromatic side chains A, B, C1 and C2. Arrows indicate the position of functionally important side chains described in the main text, left-to-right: pre-Loop B threonine in 5-HT_3_Rs; post-Loop B side chain in nAChRs; Loop C threonine in GlyRs, GABA_A_Rs, and GluCls; Loop D aromatic side chain in nAChRs; Loop D arginine/glutamine side chain in GluCls/AChBP; and Loop G arginine in GluCls. Performed in ClustalW2 (Larkin et al., 2007).

Below, the molecular determinants of agonist recognition by Cys-loop receptors will be reviewed in detail, but given the diversity of the family, generalizing detailed findings to the whole family can be confusing. Therefore, we will first point out a few noteworthy trends that we hope illustrate the relation between the various Cys-loop receptors and also distinguish Cys-loop receptors from other proteins. Given that certain Cys-loop receptors are gated by glycine, while others are activated by the chemically and structurally very different serotonin, there must naturally be a substantial degree of divergence at critical agonist-binding side chains in the agonist-binding site. Not surprisingly then, most of the ECD side chains that are *absolutely* conserved lie *outside* of the agonist-binding loops (Hibbs and Gouaux, 2011), e.g., in the eponymous Cys-loop that is situated between the ECD and the membrane-spanning domain, where it transduces conformational changes from the agonist-binding site to the channel (Kash et al., 2003; Grutter et al., 2005). Only two side chains/motives within agonist-binding loops are conserved across all Cys-loop receptors: a tryptophan in Loop D, and a Trp-X-Pro (“W-X-P”) motif in Loop A (Figure 3), which at least in 5-HT_3_Rs is known to contribute to structural integrity (Deane and Lummis, 2001). Although not absolutely conserved, a handful of ECD positions are occupied by structurally similar side chains in the vast majority of Cys-loop receptor isoforms, including aromatic side chains in Loop A, Loop B, and Loop C (Figure 3). These aromatic side chains are widely acknowledged as an “aromatic box” that surrounds the amine or ammonium nitrogen atom of most Cys-loop receptor agonists (Galzi et al., 1990; Zhong et al., 1998; Beene et al., 2004; Pless et al., 2011; Lummis et al., 2012). To avoid confusion when comparing numerous receptors, we will refer to these aromatic side chains with the three-letter amino acid abbreviation followed by the letter of the possessing loop. For example, Trp149 of the mouse α nAChR, Trp143 of the *L. stagnalis* AChBP and Phe159 of the human α1 GlyR isoforms will be referred to as TrpB, TrpB, and PheB, respectively.

**Figure 4:**
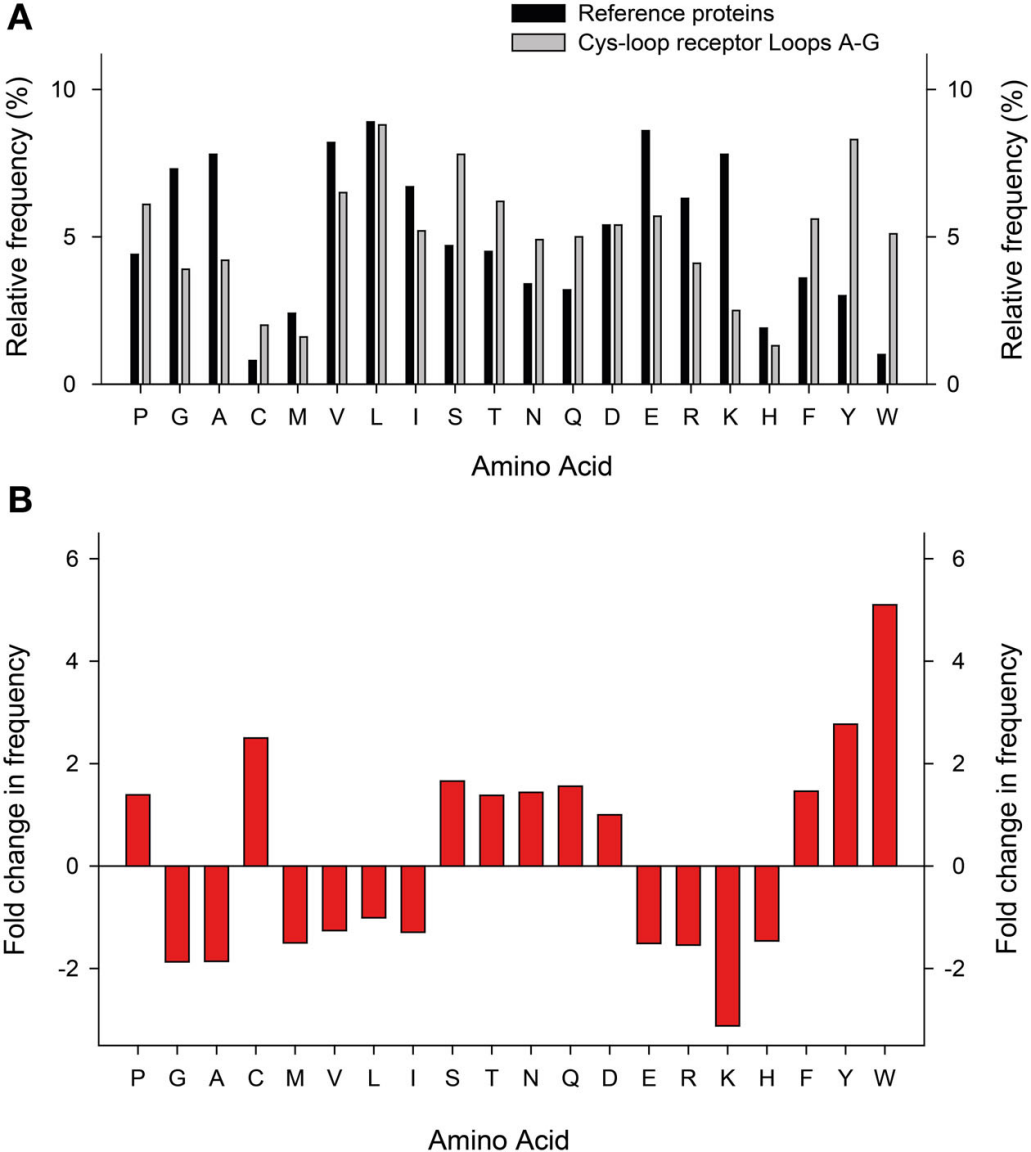
**Relative abundance of amino acids in Cys-loop receptor agonist-binding loops. (A)** Relative frequency (*y-axis*) at which each amino acid (*x-axis*) appears in a set of 520 proteins (dark columns; Brooks et al., 2002) and in the 25 Cys-loop receptor agonist-binding loops as shown in Figure 3 (light columns). **(B)** Fold increase (positive numbers) or decrease in frequency at which amino acids appear in Cys-loop receptor agonist-binding loops compared to the general protein set.

When compared to a representative number of other proteins, perhaps the most striking pattern to emerge from the amino acid composition of Cys-loop receptor ECDs is the overrepresentation of aromatic side chains (Figure 4). Especially the relative abundance of Tyr and Trp is strongly increased, which is likely due to the manifold suitability of these side chains for molecular recognition: these large amphipathic side chains can form non-polar, H-bonding, and cation-π interactions (Koide and Sidhu, 2009). This is borne out in several experimental observations that will be discussed below. Furthermore, it is noteworthy that Arg is more prevalent than Lys, which is by far the most underrepresented of all 20 side chains in Cys-loop receptor binding sites (Figure 4). A likely explanation for this observation is the fact that Arg side chains retain their positive charge even in very hydrophobic environments (Harms et al., 2011), whereas Lys side chains undergo large pK_a_ shifts depending on the dielectric of their environment (Isom et al., 2011). The reliance of agonist recognition on Arg side chains is exemplified by the Loop D and E Arg side chains of anion-selective Cys-loop receptors (see below and Figure 3). Indeed, dividing receptor subtypes according to their ion selectivity (i.e., according to their membrane-spanning domains) reveals further patterns in the ECD. Viewed in this way, the Loop A W-X-P motif is extended to a W-X-P-D-I/V motif in cation-selective receptors such as nAChRs and 5-HT_3_Rs and to W-X-P-D-T-F-F-X-N in most anion-selective receptor subunits (Figure 3). The crucial roles in agonist recognition of these motifs, although subtly different in cation- and anion-selective receptors, will be described below. At this point, we simply wish to emphasize that amino acid sequence identity can provide clues on agonist recognition. As observed by others, if chloride channel isoforms that recognize diverse agonists group together in phylogenetic analyses, it is likely that the molecular changes that lead to divergent agonist recognition are subtle and therefore identifiable (Putrenko et al., 2005; Kehoe et al., 2009; Beech et al., 2013).

## Chemical and structural insights into agonist recognition: excitatory receptors

**Figure 5:**
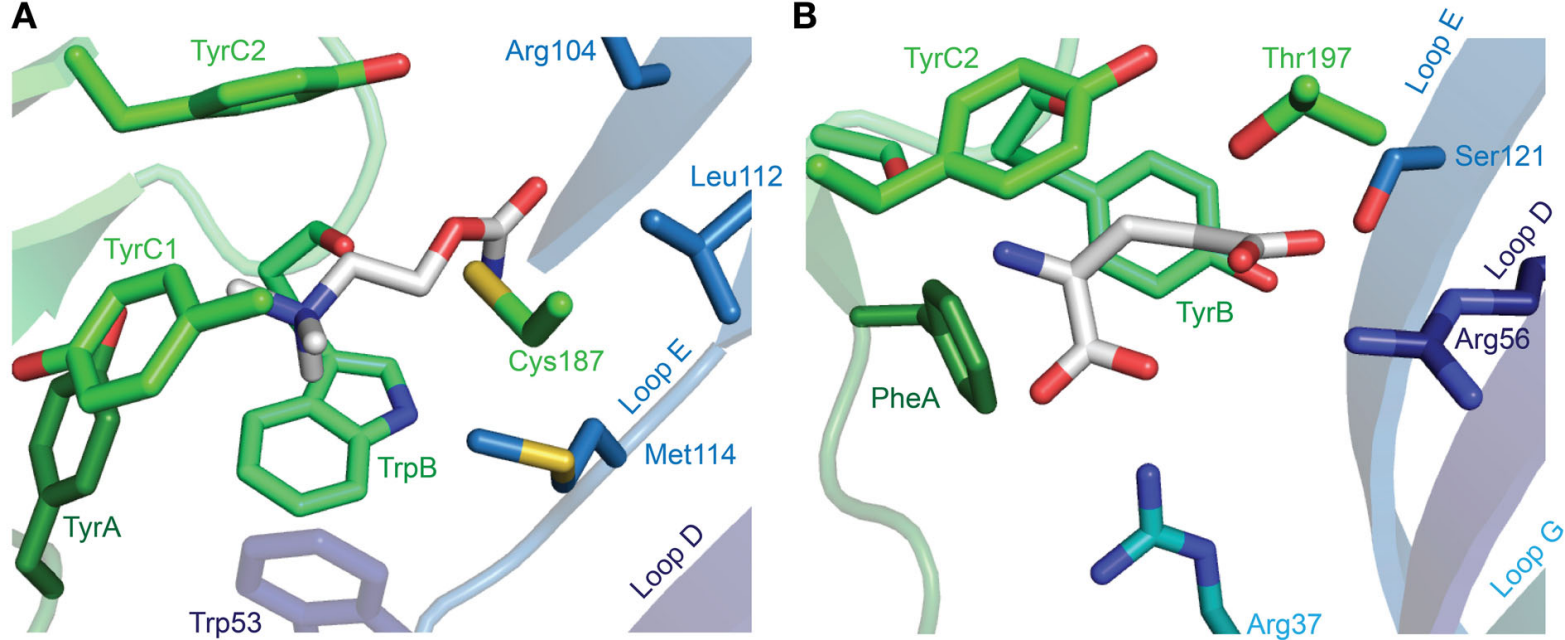
**X-ray crystallographic structures of agonist-bound receptors. (A)** *L. stagnalis* AChBP in complex with carbamylcholine (PDB entry 1UV6). **(B)** *C. elegans* α GluCl (GLC-1) in complex with glutamate (and ivermectin in the transmembrane domain; PDB entry 3RIF). In each illustration, numerous segments have been removed for clarity, including Loop C, of which only selected side chains are visible.

After the labeling of nAChRs with photo-reactive ligands identified the aromatic box (Dennis et al., 1988; Galzi et al., 1990), the field began to address the exact arrangement of the bound agonist and the nature of interactions that determine agonist recognition (Dougherty and Stauffer, 1990). This has conventionally employed the alteration of receptor structure by site-directed mutagenesis and the measure of function by electrophysiology or by radioactive ligand binding. Such experiments have been insightful, especially in the absence of high-resolution structures, showing for example that in α(2)βγδ nAChRs, the TyrA and TyrC1 hydroxyls, and the TyrC2 phenyl ring are important to the recognition of quaternary ammonium agonists by α isoforms (Tomaselli et al., 1991; O'Leary and White, 1992; Sine et al., 1994). There was some wait until the X-ray crystallographic structures of the *Lymnaea stagnalis* AChBP in complex with carbamylcholine or nicotine provided a structural explanation for these findings (Celie et al., 2004), showing on the one hand this highlighted the proximity of TyrA and TyrC1 hydroxyls to the quaternary ammonium (or other polar side chains) and, on the other hand, the sandwiching of the quaternary ammonium by TrpB and TyrC2 phenyl rings (Figure 5A). Regarding the carbamyl terminal of the agonist, the AChBP-carbamylcholine crystal structure shows the carbamyl group some three to five Å from Loop E hydrophobic side chains and a highly conserved Loop D aromatic side chain (tryptophan in AChBP; Figure 5A). Mutation of the loop D tryptophan decreases agonist affinity for α7 nAChRs (Corringer et al., 1995) and 5-HT_3_Rs (Spier and Lummis, 2000), and only in α nAChR isoforms, which form exclusively principal agonist-binding faces, is this side chain non-aromatic (Figure 3). Two principals of agonist recognition that had remained obscured from the conventional mutagenesis approach were elucidated by this structural approach: the crystal structures suggested a hydrogen bond between the backbone carbonyl of TrpB—which cannot be substituted using conventional site-directed mutagenesis—and the nicotine pyrrolidine nitrogen; and a water-bridged hydrogen bond between backbone carbonyls from Loop E and the nicotine pyridine nitrogen (Celie et al., 2004; nicotine binding is described in detail under *Other Notable Agonists* below). Agonist recognition via backbone carbonyls and water molecules suggests a tolerance for different side chains at functionally important positions and goes some way to explaining the small number of absolutely conserved ECD amino acids.

These functional and structural data collectively suggest, but do not provide direct chemical evidence, for the types of interactions that mediate agonist recognition. Such a “chemical-scale” view of agonist recognition requires the insertion of artificial amino acids (Dougherty, 2008). By exchanging single atoms or functional groups of amino acids it is possible to dissect different physico-chemical properties with atomic precision (Pless and Ahern, 2013). Via substitution of α nAChR TrpB with Trp analogs possessing a fluorinated indole ring, for example, it is possible to progressively disperse π electrons from the face of the aromatic and incrementally impair binding to cations; this approach identified a cation-π interaction between TrpB and the positively charged quaternary ammonium of acetylcholine (Zhong et al., 1998). In the same way, cation-π interactions with TrpB have also been shown for acetylcholine and nicotine in α4 nAChR isoforms (Xiu et al., 2009; Puskar et al., 2011). However, this interaction is not uniquely predicted by the presence of TrpB, as in the closely-related muscle-type α nAChR isoforms, TrpB does not form a cation-π interaction with nicotine (α nAChR isoforms lack an α4-like lysine side chain downstream of TrpB, which effects Loop B/Loop C proximity and therefore the orientation of TrpB Grutter et al., 2003; Xiu et al., 2009). Similarly, despite the absolute requirement of TrpB in homomeric α7 nAChRs for agonist recognition (Williams et al., 2009), it is only TyrA of the α7 nAChR that forms a cation-π interaction with acetylcholine (Puskar et al., 2011). Using unnatural amino acids, receptor backbone carbonyls are also amenable to modification. In α4-containing nAChRs, the replacement of the subsequent amino acid with its α-hydroxyl analog substitutes the α4 nAChR TrpB backbone carbonyl for a hydroxyl, a poorer H-bond acceptor, and selectively reduces nicotine affinity (Xiu et al., 2009). This is consistent with the H-bond suggested by structural data and homology modeling (Celie et al., 2004; Talley et al., 2006). Finally, a conserved aspartate in Loop A was initially assigned an indirect role in agonist recognition as an acceptor of H-bonds with backbone amides of Loop B (Celie et al., 2004; Lee and Sine, 2004); a subsequent study using unnatural neutral Asp derivatives in Loop A and α-hydroxyl amino acids in Loop B instead pointed to a redundant network of hydrogen bonds that does not necessarily require H-bond accepting at this Loop A position (Cashin et al., 2007).

The binding of serotonin in the 5-HT_3_R ECD is similar to that of acetylcholine in the nAChR, in that the amine nitrogen of serotonin forms a cation-π interaction with TrpB (Beene et al., 2002) and the polar 5-hydroxyl is likely oriented toward the complementary face, according to mutagenesis and homology modeling (Beene et al., 2004). This arrangement is supported by the crystal structure of a serotonin-bound *Aplysia californica* AChBP with substitutions for 5-HT_3_R-equivalent amino acids that enhance serotonin binding (Kesters et al., 2013). This structure also shows proximity of the amine nitrogen to the TyrA hydroxyl (asparagine in 5-HT_3_R) and to the TyrC2 phenyl ring (conserved in AChBP and 5-HT_3_R), consistent with the direct but distinct role of these tyrosine side chains implied by mutagenesis studies (Sine et al., 1994; Beene et al., 2004). It also suggests a water-mediated H-bond between the complementary face and the agonist hydroxyl, as well as an H-bond between the agonist amine and TrpB backbone carbonyl (Kesters et al., 2013). Thus, it is only a few differences that confer on the 5-HT_3_R its selective recognition of serotonin, including a Loop A glutamate (asparagine in nAChRs) and a pre-Loop B threonine (lysine in most nAChRs and AChBPs; Figure 3), perhaps in combination with a longer Loop C (Kesters et al., 2013).

## Chemical and structural insights into agonist recognition: inhibitory receptors

GABA_A_Rs and GlyRs, along with their inhibitory receptor cousins that are gated by various other agonists, possess TyrB or PheB side chains in place of the TrpB characteristic of excitatory receptors (Figure 3), potentially reducing the overall size of the agonist-binding site so as to optimize it for binding smaller and sterically less constrained agonists such as glycine and GABA. Nonetheless, cation-π interactions between the amino groups of glycine or GABA have been demonstrated for PheB in the α1 GlyR (Pless et al., 2008), TyrB in the ρ GABA_A_R (Lummis et al., 2005) and PheB in the insect RDL GABA receptor (Lummis et al., 2011). In MOD-1 (serotonin-gated) and RDL inhibitory receptors, cation-π interactions involving TrpC2 or TyrC2 (also) occur (Mu et al., 2003; Lummis et al., 2011). This perhaps reiterates that the structure of the agonist-binding site and the binding mode of various agonists are determined not only by ECD amino acid identity but also by length of loops and the orientation of side chains outside of the agonist-binding site (Liu et al., 2005; Kehoe et al., 2009; Xiu et al., 2009). Indeed, Loop A has been proposed as the main determinant of the different agonist recognition by α and β GlyR isoforms (Shan et al., 2012), yet their loop A sequences are 100% identical.

The glutamate-bound *C. elegans* α GluCl crystal structure provides the first high-resolution data on an inhibitory, and indeed on a full-length eukaryotic, Cys-loop receptor (Hibbs and Gouaux, 2011). It shows that the amine nitrogen of glutamate interacts with two backbone carbonyls from Loop B (TyrB and the preceding serine) from its position between three aromatic side chains, PheA (Phe91), TyrB (Tyr151), and TyrC2 (Tyr200; there is no C1 aromatic in the GluCl). The functional importance of the C2 aromatic is evident in reduced responses to agonists upon the mutation of TyrC2 in the *C. elegans* β GluCl (Li et al., 2002; also called GLC-2; Beech et al., 2010), PheC2 in α or TyrC2 in β isoforms of GlyRs (Grudzinska et al., 2005), TyrC2 in the β isoform of heteromeric GABA_A_Rs (Amin and Weiss, 1993) and TyrC2 in insect RDL GABA receptors (Lummis et al., 2011). The manner in which this side chain interacts with the agonist amine differs across receptors, however. In *C. elegans* MOD-1 (Mu et al., 2003), in *Drosophila* RDL (Lummis et al., 2011) and in GABA recognition by the bacterial ELIC (Spurny et al., 2012), aromatic C2 forms a cation-π interaction, whereas in vertebrate GlyRs (Pless et al., 2008) and GABA_A_Rs, (Lummis et al., 2005; Padgett et al., 2007) this is not the case. From a structural perspective, PheA is positioned similarly to TyrA (Tyr89) in AChBP (Figure 5), and could serve a similar function in stabilizing agonist nitrogen atoms, but despite their similar arrangement in space, inhibitory receptor PheA and excitatory receptor TyrA differ significantly in two ways: firstly, the phenylalanine at this position in most inhibitory receptor isoforms is devoid of H-bonding ability, whereas the hydroxyl group of Tyr93 (α), Tyr97 (α4), and Tyr92 (α7) in vertebrate nAChR isoforms is (also) crucial for acetylcholine recognition (Sine et al., 1994; Puskar et al., 2011). Secondly, its position in inhibitory receptor isoforms is actually two positions upstream of that in nAChRs (Figure 3). This shows that inhibitory and excitatory receptors have arrived at a similar structural arrangement via different molecular pathways, highlighting both the importance of X-ray crystollagraphic studies and the independent evolution of agonist recognition within the two classes of receptors. Interestingly, the only inhibitory receptor isoforms to have incorporated an aromatic side chain two positions downstream of PheA are the α1-α5 GABA_A_R isoforms (Figure 3), which contain a histidine in this location. It is this histidine that determines the high affinity of benzodiazepines for α1-α5-containing GABA_A_Rs (Wieland et al., 1992).

The above illustrates that the amine of most inhibitory receptor agonists is accommodated by the aromatic box on the principal face of the ECD, much like excitatory receptor agonists. However, at the complementary face of the agonist-binding site, the charged carboxyl group of glycine and GABA diverges considerably from the acetyl, carbamyl, or hydroxyl groups of acetylcholine, carbamylcholine, and serotonin, respectively. The α GluCl crystal structure provides a logical explanation for this difference. The positively charged guanidine side chain of Arg56 in Loop D is within 3 Å of the γ carboxyl of glutamate (Hibbs and Gouaux, 2011; Figure 4B), suggesting a charge/charge interaction. This Loop D arginine is present in numerous GlyR, GABA_A_R and GluCl isoforms (some shown in Figure 3), each of which contributes the complementary face to the agonist-binding site of functional receptors (Ffrench-Constant et al., 1993; Cromer et al., 2002; Bamber et al., 2003; Grudzinska et al., 2005; Goldschen-Ohm et al., 2011). In vertebrate GABA_A_Rs and GlyRs, substitution of this arginine for alanine drastically reduces agonist sensitivity (Grudzinska et al., 2005; Goldschen-Ohm et al., 2011; it has not been substituted/tested in GluCls). Notably, this Loop D arginine is absent from HisCl1, ACC1, MOD-1, and LGC-55, isoforms that form inhibitory receptors for biogenic amines carrying no negative charge. In these isoforms, this Loop D position is instead occupied by polar amino acids that could foreseeably interact with the hydroxyl termini of these agonists directly or through water molecules, as observed in excitatory receptors. GABA_A_R β isoforms, which contribute only the principal face to GABA binding (Cromer et al., 2002), also lack this arginine (Figure 3). The hydroxyl of a Loop C threonine side chain also appears to interact with the γ carboxyl (Figure 4B), and mutation of this threonine (Figure 3) to side chains devoid of hydroxyl groups severely impairs agonist recognition in GlyRs and GABA_A_Rs (Vandenberg et al., 1992; Amin and Weiss, 1993, 1994).

In addition to the γ carboxyl, glutamate also contains an α carboxyl, constituting a second negative charge on the agonist. According to the GluCl crystal structure, the latter interacts with the positively charged side chain of a Loop G arginine (Hibbs and Gouaux, 2011), whose substitution for alanine in the β GluCl reduces glutamate sensitivity (Li et al., 2002). (α GluCl isoforms form homomers that bind glutamate Cheeseman et al., 2001; Frazier et al., 2013 but are not readily gated by glutamate alone Cully et al., 1994). This Loop G arginine is absent from GABA_A_R and GlyRs (Figure 3) that need only accommodate a single agonist carboxyl. Thus, the close relation of ECD sequences in GABA_A_Rs, GlyRs, and GluCls, together with structural and functional data, suggests that inhibitory receptor agonists share a similar binding mode, with an amine terminal surrounded by the principal face and a carboxyl terminal interacting with the complementary face.

## Other notable agonists

**Figure 6:**
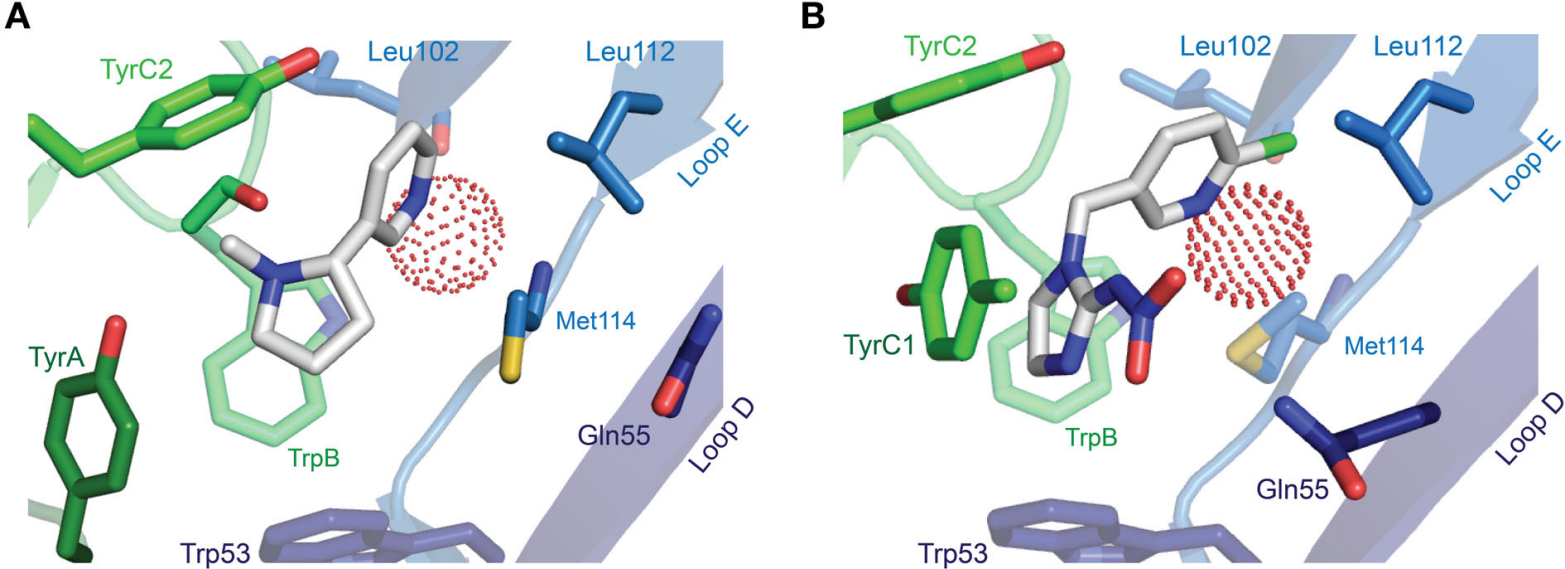
**X-ray crystallographic structures of other notable agonists. (A)** *L. stagnalis* AChBP in complex with nicotine (PDB entry 1UW6). **(B)** *L. stagnalis* AChBP in complex with imidacloprid (PDB entry 2ZJU). A red sphere illustrates the oxygen atom of a water molecule that bridges agonist pyridines to backbone Leu102 carbonyl and Met114 amide groups. In each illustration, numerous segments have been removed for clarity, including Loop C, of which only selected side chains are visible.

The description of agonist binding has so far focused on a handful of endogenous transmitters, but the binding of numerous other relevant agonists has been studied, some of which are isoform-selective (and used in dissecting isoform composition), and some of which are potent neurotoxins or widely used pharmaceuticals. Nicotine activates nAChRs more potently than acetylcholine itself (Chavez-Noriega et al., 1997), and its activation of these receptors stimulates reward pathways in the brain (Salminen et al., 1999) and up-regulates nAChR expression (Sallette et al., 2005). The crystal structure of nicotine-bound AChBP shows nicotine in a similar position to carbamylcholine (Celie et al., 2004), with the pyrrolidine coordinated by principal face aromatic side chains and the pyridine oriented toward the complementary face (Figure 6A). Several nicotine-like compounds are utilized as inseciticides (Millar and Denholm, 2007), because they activate insect nAChRs more potently than vertebrate nAChRs (Tomizawa and Casida, 2005). Binding of the canonical neonicotinoid, imidacloprid, has been illustrated by X-ray crystallography (Ihara et al., 2008; Talley et al., 2008), and reminiscent of nicotine, the imidazole nitrogens are surrounded by the aromatic box, and the chloropyridine “tail” points toward Loop E (Figure 6B). The additional nitramide tail, pointing toward a Loop D glutamine in AChBP, likely contributes to the selectivity of imidacloprid for insect nAChRs, as mutation of this glutamine in the chick α7 nAChR to the arginine present in insect α nAChR isoforms increases imidacloprid potency (Shimomura et al., 2002). In a curious parallel with inhibitory receptors, the imdacloprid nitramide/Loop D arginine interaction of insect acetylcholine receptors thus structurally reflects the glutamate carboxyl/Loop D arginine interaction at Loop D of inhibitory receptors (compare Figures 5B, 6B; AChBP Gln55 corresponds to α GluCl Arg56). Decreased imidacloprid-sensitivity in a naturally occurring Tyr151Ser mutation in a planthopper nAChR (Liu et al., 2005) provides another example of allosteric control of the agonist-binding site, as this side chain (histidine in *L. stagnalis* AChBP) is two positions downstream of TrpB and oriented well away from the bound imidacloprid molecule (Ihara et al., 2008; Talley et al., 2008).

Several nAChR agonists are also lethal to roundworms, exemplified by the well-established anthelmintics levamisole and pyrantel (Austin et al., 1966; Thienpont et al., 1966) and the novel anthelmintic monepantel (Kaminsky et al., 2008), which each act on roundworm nAChRs in a similar manner as the endogenous agonist acetylcholine (Harrow and Gration, 1985; Robertson et al., 1994; Rufener et al., 2010). The diversity and stoichiometry of nematode acetylcholine receptor isoforms (few of which can truly be referred to as “nicotinic”) is substantial, and the exact make-up of the native binding sites for these agonists remains enigmatic (Martin et al., 2012). Such sites have not been probed by mutagenesis or structural methods, but altered agonist selectivity upon selective over-expression of particular isoforms may provide some hints as to the molecular determinants of agonist recognition (Ballivet et al., 1996; Raymond et al., 2000; Williamson et al., 2009; Boulin et al., 2011). Notably, the levamisole sensitivity of UNC-29 and UNC-38 nAChR isoforms seems to depend on a glutamate side chain at the position four downstream from TrpB (Rayes et al., 2004), perhaps indicating that, like other agonists, levamisole recognition is sensitive to Loop B-Loop C interactions.

Given the region-specific and behavior-specific expression of GABA_A_Rs in the brain (Rudolph et al., 1999; Low et al., 2000) and the potency with which GABA_A_R agonists depress neuronal function (Krogsgaard-Larsen and Falch, 1981), much effort has been dedicated to the development of subtype-selective GABA_A_R agonists. The structural analogy these compounds share with GABA, together with mutagenesis data, suggest that the molecular determinants of recognition are similar to those outlined above for GABA (Abdel-Halim et al., 2008). Certain agonists, such as muscimol and 4,5,6,7-tetrahydoisoxazolo[5,4-c]pyridin-3(2H)-one (THIP), show some subtype-selectivity (Petersen et al., 2013), possibly due to the differential influence of these agonists on subsequent conformational changes in different isoform combinations (Mortensen et al., 2010). This in turn might be a consequence of slightly different binding modes from GABA, as muscimol and THIP possess hydroxyl termini in place of the carboxyl of GABA, perhaps allowing H-bonds with and water bridges to the complementary face (Bergmann et al., 2013), as opposed to the charge/charge interactions proposed for GABA.

## Recurring themes of agonist recognition in cys-loop receptors and future challenges

The preceding sections have highlighted a few principles that are common to the family: many—if not all—receptors form a strong cation-π interaction between an aromatic side chain in the binding site and the agonist protonated amine (or ammonium); for agonists with one or more carboxyl groups, the negative charge is likely accommodated by charge/charge interactions with one or more arginine side chains; and in the case of receptors for biogenic amines, which lack a negative charge, the latter interaction appears to be compensated by H-bonds. The striking reliance of Cys-loop receptors on cation-π interactions with agonists raises the question of why this interaction is preferred over charge/charge interactions (such as those predicted for the carboxyl groups). We propose the following reasons for this observation. First, the cation-π interaction is energetically less dependent on the surrounding dielectric environment (Gallivan and Dougherty, 2000), which is crucial given that the latter changes dramatically during the agonist-induced closure of the binding pocket (Wagner and Czajkowski, 2001; Hansen et al., 2005; Sharkey and Czajkowski, 2008; Pless and Lynch, 2009; Sauguet et al., 2014); Lys, Asp and Glu side chains, on the other hand, can undergo drastic changes in their protonation state depending on the dielectric environment (Isom et al., 2008). Furthermore, and unlike charge/charge interactions, the cation-π interaction requires stringent geometrical constraints and can only occur within a narrow window of angles of an *en face* interaction between the π electron cloud and a cation (Gallivan and Dougherty, 1999). This may help to increase selectivity and further aid the precise orientation of the agonist in the binding site. On the one hand, it is hard to conceive how the related receptor subtypes could have developed such high specificity for different agonists. On the other, the findings summarized in this review show that agonist recognition in Cys-loop receptors requires a complex molecular orchestration of side chains, backbone carbonyls, and waters—both inside and outside the actual binding site. Finally, the wide array of possibilities regarding distinct subunit interfaces (and thus distinct binding pockets) further contributes to the diversity and complexity of agonist recognition in this receptor family.

Despite the intense research that has focused on these receptors for decades, crucial questions remain as to the precise role of some of the loop structures in agonist-recognition. For example, Loop F plays a direct role in agonist recognition according to studies on nAChRs and 5HT_3_Rs, and heteromeric GABA_A_Rs (Corringer et al., 1995; Newell and Czajkowski, 2003; Thompson et al., 2006), but not according to ρ-type GABA_A_R studies (Sedelnikova et al., 2005; Khatri et al., 2009). As mutations at different positions in Loop F preferentially affect agonist affinity in different states of the activation process, Loop F-agonist interactions probably change substantially during ligand-induced activation (Sine et al., 2002), and this is not yet explicable by available structural data. Further, and despite some recent progress (reviewed in Nys et al., 2013), our understanding of which molecular determinants discriminate between full and partial agonists is largely unexplored. However, a better understanding of these factors will be essential to fully understand ligand recognition and gating in Cys-loop receptors. Finally, a spectacular example demonstrating how much there is still to be learnt about even basic principles of agonist recognition in Cys-loop receptors was recently published by Stornaiuolo et al. (2013). Their study demonstrated that the canonical acetylcholine-binding site of AChBP can accommodate three copies of an aromatic small molecule in an ordered π−π stack (three identical molecules per binding site), a rare example of supramolecular binding at a canonical binding site. These and other studies will no doubt continue to expand our knowledge about how these therapeutically relevant receptors recognize and bind their agonists.

### Conflict of interest statement

The authors declare that the research was conducted in the absence of any commercial or financial relationships that could be construed as a potential conflict of interest.
